# Association of total testosterone levels with cardiometabolic diseases in men with erectile dysfunction

**DOI:** 10.1093/sexmed/qfae089

**Published:** 2025-01-10

**Authors:** Bing-Tau Chen, Ping-Ju Tsai, Bang-Ping Jiann

**Affiliations:** Division of Urology, Department of Surgery, Kaohsiung Armed Forces General Hospital, Kaohsiung City 80284, Taiwan, Republic of China; Visiting staff, Division of Urology, Department of Surgery, Yuan's General Hospital, Kaohsiung City 802793, Taiwan, Republic of China; Visiting staff, Division of Urology, Department of Surgery, Yuan's General Hospital, Kaohsiung City 802793, Taiwan, Republic of China

**Keywords:** total testosterone, erectile dysfunction, obesity, cardiometabolic disease, diabetes mellitus

## Abstract

**Background:**

Both serum testosterone (T) levels and erectile dysfunction (ED) are associated with systemic diseases in men and ED is the most common presenting symptom of hypogonadism.

**Aim:**

To evaluate the association of serum total testosterone (TT) levels with cardiometabolic diseases in men with ED.

**Methods:**

Serum endogenous TT levels were determined to evaluate their associations with cardiometabolic diseases in men with ED in outpatient clinics. Participants were divided into hypogonadal with TT < 350 ng/dL (12.1 nmol/L) and eugonadal groups, as well as into four equal quartiles based on TT levels. The Framingham risk score was used to estimate individual 10-year coronary heart disease (CHD) risk.

**Main Outcome Measures:**

Cardiometabolic factors included obesity, diabetes mellitus (DM), hypertension (HT), dyslipidemia, and the Framingham risk score.

**Results:**

From 2010 to 2021, a total of 4467 subjects with ED were consecutively recruited for this study, and 3909 subjects’ (87.5%) data with a mean age of 53.0 ± 12.9 (20.0–88.0) years had data eligible for analysis. Testosterone levels declined with age and a higher body mass index (BMI) was associated with lower T levels across all age groups (*P* < .001). Compared to the eugonadal group, the hypogonadal group was older and had a higher BMI and more cardiometabolic diseases (all *P* < .01). In multivariate analysis, odds ratio (OR) for hypogonadism was highest in men with obesity (2.51), followed by age group of ≥70 years (2.32), DM (1.59), HT (1.41), and dyslipidemia (1.26). Compared with the lowest TT quartile, higher quartiles of TT had significantly lower risk for cardiometabolic diseases (all *P* < .001). Among men over 50 yrs, hypogonadal men had a higher 10-year CHD risk than eugonadal men as predicted by the Framingham risk score (*P* < .001).

**Clinical implications:**

Our results highlight the value of determining TT levels in men with ED because of their association with cardiometabolic diseases and the potential benefits of T therapy for improving men’s health.

**Strengths and Limitations:**

Strengths of this study include a relatively large sample and detailed medical history collection. Limitations included a small portion of subjects with repeat TT tests, and the lack of data on free T and bioavailable T levels, and single-site recruitment.

**Conclusions:**

TT levels are independently associated with cardiometabolic diseases including obesity, DM, HT, and dyslipidemia, and indicate a higher risk for CHD in men with ED. Measuring TT levels in men with ED presents an opportunity to improve overall health and reduce CV risk.

## Introduction

Erectile dysfunction (ED) is a common medical disease, characterized by the recurring or persistent inability of a man to achieve and/or maintain a penile erection adequate for satisfactory sexual intercourse.^1^ Men with ED are associated with older and are associated with major adverse cardiovascular events (MACE), hypertension (HT), dyslipidemia, diabetes mellitus (DM), depression, premature ejaculation, and sedentary lifestyle.^2–4^ Approximately 60% of Taiwanese men with ED have been diagnosed with at least one chronic disease,^5^ attributed to shared pathophysiological mechanisms.^4^ ED is primarily recognized as a vascular disease and is commonly found in men with known cardiovascular disease (CVD).^6^ The severity of ED strongly correlates with severity of CVD, and recent studies suggest ED may serve as a sentinel marker for occult CVD.^7^ The onset of ED precedes the development of coronary artery disease (CAD), stroke, and peripheral artery occlusion disease by 2–5 years.^8^ The artery size hypothesis explained this temporal sequence, as the penile artery’s small diameter (1–2 mm) compared to the coronary artery (3-4 mm) makes it more susceptible to occlusion.^9^ Pathological mechanisms such as endothelial dysfunction, impaired smooth muscle contractility regulation, autonomic neuropathy, androgens, and metabolic factors link ED with CAD.^10^ It is recommended men with ED be counseled regarding its association with CVD and other health conditions warranting evaluation and treatment.^11^ Diagnosis of ED provides a window of opportunity to aggressively reduce CVD risk, especially in younger men (30–60 years) who are at substantially increased risk.^12^

Testosterone (T) is the most important androgen in men. Most circulating T (98%) is bound to sex-hormone binding globulin (SHBG) (60%) or albumin (38%) with approximately 2% remaining unbound (free T). Bioavailable T levels, which indicate the amount the human body can utilize, are the sum of free T and albumin-bound T. Hypogonadism or T deficiency (TD) defined as a clinical syndrome arising from inadequate production of T due to disruption at one or more levels of the hypothalamic–pituitary-testicular axis.^13^ Diagnosis requires clinical identification of relevant symptoms and laboratory confirmation of low T levels. Symptoms of TD include sexual symptoms (reduced libido, ED, difficulty achieving orgasm) and non-sexual symptoms (fatigue, lack of energy, decreased vitality, depressed mood, and reduced motivation).^14^ The cut-off value of total testosterone (TT) for TD diagnosis is 350 ng/dL (12.1 nmol/L) according to the International Society of Sexual Medicine (ISSM)^15^ and 300 ng/dL (10.4 nmol/L) per the American Urological Association (AUA).^16^ At least two morning serum TT measures are recommended for TD diagnosis.^16^ Prevalence of male hypogonadism varies widely, ranging from 2.1% to 38.7%, due to differences in diagnostic criteria and study participants.^13^^,^^14^^,^^17^ Regardless of the cause, TD’s clinical effects are common, although they may vary in severity or based on the age of onset.^18^ In addition to contributing to obesity, TD may result in dyslipidemia, characterized by increased levels of total cholesterol, low-density lipoprotein cholesterol, and triglycerides.^19^ In middle-aged men, TD indicates early progression to metabolic syndrome and DM.^20^ Increasing evidence suggests low T levels are associated with an elevated risk of CVD.^21–25^

ED is often the most common complaint among men with TD and is considered the endocrine disorder most closely related to T levels.^26^^,^^27^ ED, TD, metabolic syndrome, and CVD are closely linked, impacting patient morbidity, and mortality.^28^^,^^29^ Routine screening for TD in asymptomatic men is not recommended.^21^ However, ISSM advises screening for TD in men with symptoms or signs typically associated with TD, particularly sexual dysfunctions.^30^

Both TD and ED are associated with medical issues, providing an opportunity to address broader men's health concerns.^4^^,^^31^ Although the association between T and cardiometabolic diseases is well established, more studies are needed to determine the clinical significance of screening T levels in men with sexual dysfunction. Herein, this cross-sectional observational study aimed to address two questions:

(1) Are endogenous TT levels associated with conventional CVD risk factors in men with ED?

(2) Are TT levels associated with CVD in this cohort, as indicated by the Framingham risk score?

## Materials and methods

Male subjects presenting to urological outpatient clinics with ED complaints were screened for serum TT levels from 2010 to 2021. Medical histories were obtained by asking, “*Have you been diagnosed with diabetes mellitus (DM), hypertension (HT), dyslipidemia, or MACE*?” Blood pressure, body weight, height, and waist circumference were measured by a nurse. Hypogonadism was defined as TT < 350 ng/dL (12.1 nmol/L) measured by Microparticle-enzyme Immunoassay (MEIA) AxSYM Testosterone reagent (Abbott). Blood sampling for TT measurement was advised to be accomplished in the morning under fasting condition. DM was defined as self-reported disease, fasting blood sugar ≥126 mg/dL, or HbA1c ≥ 6.5%. Dyslipidemia was defined as self-reported disease or abnormal lipid profiles. Body mass index (BMI) categories were as follows: obesity (≥ 27 kg/m^2^), overweight (24 ≤ BMI < 27), and normal (< 24).

The severity of ED was assessed by the Sexual Health Inventory for Men (SHIM) whereas ED was defined as a total score < 22, mild ED as 17 –21, mild to moderate ED as 12–16, moderate ED as 8–11, and severe ED as <8.

To evaluate TT levels’ impact on cardiometabolic diseases, participants were divided into four equal quartiles based on TT levels, with the lowest quartile serving as the reference group. The Framingham risk score was used to predict the 10-year cardiovascular (CV) event risk based on variables such as sex, age, blood pressure, lipid levels, anti-hypertensive medication use, smoking status, and DM.^32^

Participants with prostate cancer, advanced malignancy, spinal cord injury, illicit drug use, or alcoholism were excluded. Written informed consent was obtained, and the study protocol was approved by the Institutional Review Board,

### Statistical methods

Descriptive data were reported as the mean ± standard deviation (range) for continuous variables and as percentages (numbers) for categorical variables. Categorical variables were compared using the Chi-Square test, while continuous variables were analyzed with unpaired Student’s t-test or two-way analysis of variance as appropriate. Simple linear regression was employed to evaluate the decline rate of TT with age. Multiple logistic regression estimated the relative odds ratio (OR) of hypogonadism and vascular risk factors, adjusting for potential confounders, The null hypothesis was rejected when a *P* value was < .05. IBM SPSS Statistics for Windows, version 26 (IBM Corp., Armonk, N.Y., U.S.) was utilized for statistical analyses. Excel 2019 (Microsoft, Redmond, W.A., U.S.) was used for data entry.

## Results

From 2010 to 2021, 4467 men presenting with complaints of ED at outpatient clinics were recruited. After excluding individuals meeting exclusion criteria (n = 93) and those lacking TT measurements (n = 465), data from 3909 participants (87.5%) were eligible for analysis. The mean age of the cohort was 53.0 ± 12.9 years (range: 20.0–88.0).

Of these, 286 (7.3%) underwent repeat TT testing, yielding mean initial and repeat TT levels of 280 ng/dL (9.7 nmol/L) and 310 ng/dL (10.7 nmol/L), respectively. Final TT levels were calculated as the average of these measures. Based on TT levels, participants were categorized into hypogonadal (31.4%, n = 1229) and eugonadal (68.6%, n = 2680) groups.

Table 1 summarizes the demographic data, comorbidities, laboratory results (fasting blood sugar, HbA1c, and lipid profiles), and erectile function comparisons between hypogonadal and eugonadal groups. The hypogonadal group was older, had a higher BMI and exhibited a higher prevalence of cardiometabolic diseases, including DM, HT, dyslipidemia, and MACE (all *P* < .05). While the hypogonadal group had a marginally lower SHIM score (10.4 ± 5.4 vs. 10.8 ± 5.3, *P* < .05), the severity of ED did not significantly differ between groups (*P* > .05).

**Table 1:** Comparison of demographic data, laboratory data, and erectile function between the groups of hypogonadal and eugonadal men with ED.

**Variables**	**Men with low testosterone levels (n = 1229)**	**Men with normal testosterone levels (n = 2680)**	***P-*value**
Age, yrs	54.9 ± 11.6 (20.0–88.0)	52.2 ± 13.4 (20.0–88.0)	*P* < .001
WC, cm	93.2 ± 10.1 (68.0–150.0)	88.7 ± 9.3 (61.0–125.0)	*P* < .001
BMI, kg/m^2^	26.7 ± 3.9 (16.8–45.3)	24.9 ± 3.4 (14.5–41.2)	*P* < .001
Smoking habit			*P* = .19
*Never*	55.0%	56.8%	
*Current smoker*	22.9%	23.9%	
*Quit smoker*	22.1%	19.3%	
DM	40.1%	24.0%	*P* < .001
HT	46.1%	29.6%	*P* < .001
Dyslipidemia	64.9%	51.7%	*P* < .001
MACE	4.8%	3.1%	*P* < .01
TC, mg/dL	186.8 ± 36.8 (85.0–352.0)	187.9 ± 36.7 (50.0–381.0)	*P* = .45
TG, mg/dL	180.2 ± 143.6 (39.0–1992.0)	135.2 ± 106.2 (25.0–1947.0)	*P* < .001
HDL, mg/dL	42.6 ± 11.8 (8.0–159.0)	46.2 ± 11.9 (12.0–161.0)	*P* < .001
LDL, mg/dL	104.9 ± 27.8 (12.0–225.0)	105.9 ± 28.7 (11.0–250.0)	*P* = .32
FBS, mg/dL	123.7 ± 51.2 (71.0–521.0)	111.2 ± 41.7 (54.0–471.0)	*P* < .001
HbA1c, %	7.2 ± 1.7 (4.7–16.5)	7.1 ± 1.9 (4.4–15.5)	*P* = .33
TT	260 ± 60 (10–350) ng/dL 9.0 ± 2.1 (0.3–12.1) nmol/L	560 ± 180 (351–1000) ng/dL 19.4 ± 6.2 (12.1–61.7) nmol/L	*P* < .001
SHIM score	10.4 ± 5.4 (1.0–25.0)	10.8 ± 5.3 (1.0–25.0)	*P* < .05
ED Severity			*P* = .42
*Severe*	29.7%	27.8%	
*Moderate*	23.4%	24.7%	
*Mild to moderate*	27.3%	29.0%	
*Mild*	10.8%	11.8%	
*Normal*	1.6%	2.2%	

Abbreviations: ED = erectile dysfunction, WC = waist circumference, BMI = body mass index, DM = diabetes mellitus, HT = hypertension, MACE = major adverse cardiovascular events, TC = total cholesterol, TG = triglycerides, HDL = high-density lipoprotein, LDL = low-density lipoprotein, FBS = fasting blood sugar, HbA1c = glycated hemoglobin, SHIM = Sexual Health Inventory for Men

**Figure 1:**
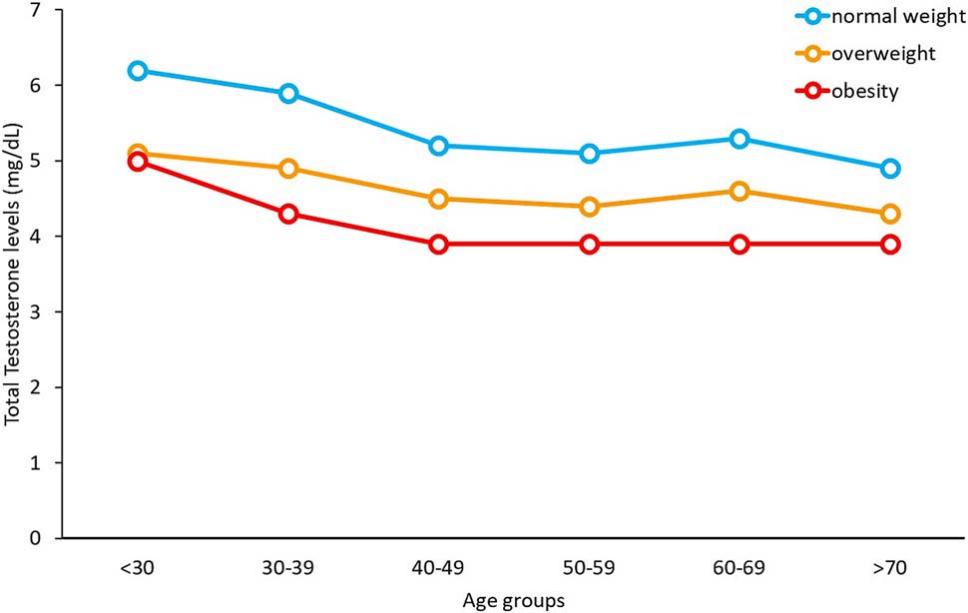
Serum total testosterone levels in different age groups and stratified by body mass index categorized as normal weight, overweight, and obesity.

TT levels declined with age, decreasing by an average decrease of 17 ng/dL (0.59 nmol/L) per decade, as demonstrated by simple linear regression mode. Figure 1 illustrates age-stratified TT levels by body weight. Testosterone decline was observed across all BMI categories (*P* < 0.05), with higher BMI associated with lower TT levels (*P* < 0.05). There was no significant interaction between age groups and BMI.

Multiple logistic regression analysis, adjusting for confounders, showed that obesity had the highest OR for hypogonadism (OR = 2.51, 95% confidence interval (CI): 2.01–3.02), followed by age group of ≥70 years (OR = 2.32, 95% CI:1.36–3.97), DM (OR = 1.59, 95% CI:1.36–1.86), HT (OR = 1.41, 95% CI: 1.20–1.64), and dyslipidemia (OR = 1.26, 95% CI: 1.08–1.46) (Table 2).

**Table 2:** Multiple logistic regression for OR of having male hypogonadism in men with ED after adjustment for other risk factors.

Variables	Prevalence of hypogonadism	*P*-value	OR of hypogonadism (95% CI)	*P*-value
Age group, yrs		< .0001		.007
20–29	12.1%		Reference	
30–39	21.4%		1.43 (.85–2.42)	.178
40–49	33.5%		2.18 (1.32–3.54)	.002
50–59	34.0%		2.03 (1.24–3.34)	.005
60–99	33.6%		1.96 (1.19–3.24)	.009
≥ 70	35.5%		2.32 (1.36–3.97)	.002
BMI, kg/m^2^				
< 24	20.3%	.000	Reference	
≥ 24 and < 27	32.0%		1.57 (1.32–1.88)	.000
≥ 27	44.8%		2.51 (2.01–3.02)	.000
DM				
No	26.5%	.000	Reference	
Yes	43.3%		1.59 (1.36–1.86)	.000
Dyslipidemia				
No	25.0%	.000	Reference	
Yes	36.5%		1.26 (1.08–1.46)	.003
HT				
No	25.6%	.000	Reference	
Yes	31.0%		1.41 (1.20–1.64)	.000

Abbreviations: OR = odds ratio, ED = erectile dysfunction, BMI = body mass index, DM = diabetes mellitus, HT = hypertension

Hypogonadal men over 50 years old had a higher 10-year CVD risk, assessed by the Framingham risk score, compared to eugonadal men (*P* < 0.05). Table 3 presents mean TT levels by quartiles, showing that higher TT levels were significantly associated with lower risks for DM, HT, and dyslipidemia (all *P* < .05).

**Table 3:** Multiple logistic regression for OR of DM, HT, and dyslipidemia stratified by TT levels after adjusting for other confounders.

**TT groups (n)**	**Mean TT levels**	**OR for obesity** **(95% CI)**	**OR for DM** **(95% CI)**	**OR for HT** **(95% CI)**	**OR for dyslipidemia (95% CI)**
TT ≤ 320 ng/dL (11.1 nmol/L) (982)	240 ± 60 (10–320) ng/dL 8.3 ± 2.1 (0.3–11.1) nmol/L	Reference	Reference	Reference	Reference
320 ng/dL (11.1 nmol/L) < TT ≤ 4.3 ng/mL (14.9 nmol/L) (979)	380 ± 30 (321–430) ng/dL 13.2 ± 1.0 (8.3–14.9) nmol/L	0.80^*^ (0.66–0.97)	0.78^*^ (0.64–0.95)	0.76^*^^*^ (0.62–0.92)	0.86 (0.71–1.05)
430 ng/dL (14.9 nmol/L) < TT ≤ 570 ng/dL (19.8 nmol/L) (974)	500 ± 40 (431–570) ng/dL 17.3 ± 1.4 (14.9–19.8) nmol/L	0.47^*^^*^ (0.39–0.58)	0.58^*^^*^ (0.47–0.71)	0.71^*^^*^ (0.58) –0.88	0.729^*^ (0.60–0.89)
TT > 570 ng/dL (19.8 nmol/L) (974)	750 ± 170 (571–1790) ng/dL 26.0 ± 5.9 (19.8–62.1) nmol/L	0.37^*^^*^ (0.30–0.46)	0.41^*^^*^ (0.33–0.51)	0.69^*^^*^ (0.55–0.85)	0.65^*^^*^ (0.53–0.79)

Abbreviations: TT = total testosterone, OR = odds ratio. DM = diabetes mellitus, HT = hypertension

^*^ *P < .*05, ^*^^*^P < .001

## Discussion

Both ED and TD are common medical conditions linked to cardiometabolic diseases and increased CV risk in men. In this study, we assessed the relationship between TT levels and cardiometabolic diseases in 3909 men with ED from outpatient clinics. Our multivariate logistic regression analysis identified obesity as the strongest predictor of hypogonadism (OR = 2.51), followed by older age, DM, and HT. These findings as well as the prevalence of TD with diseases align with the Hypogonadism in Men (HIM) study,^17^ despite differences in TT cut-off levels and participant age criteria.

Obesity—recognized as a major predictor of male hypogonadism^17^^,^^19^—is linked to hypogonadism via a bidirectional relationship.^33^ Many causative and correlative factors existed on both sides of the equation.^17^ Male obesity-associated secondary hypogonadism (MOSH) involves increased levels of leptin, insulin, proinflammatory cytokines, and estrogen, which contribute to functional hypogonadotropic hypogonadism.^34^ The diagnosis of MOSH requires low T levels and the presence of the non-specific features of hypogonadism, including tiredness, lethargy, and physical weakness.^35^ A detailed drug history and comprehensive hormonal survey, including sex, thyroid, and adrenal gland hormones are very important to exclude alternative causes of MOSH. Lifestyle modifications, including weight reduction, form the cornerstone of MOSH management. Notably, weight loss 5 kg can increase TT levels by approximately 30 ng/dL (1.0 nmol/L) on average and possibly can revert hypogonadism in men.^36^ However, achieving sustainable weight loss remains challenging.^36^ Testosterone therapy (TTh) can supplement MOSH treatment by reducing fat mass, increasing lean body mass, and improving metabolic profiles, including insulin resistance (IR), blood pressure, and lipid profiles.^37–40^ TTh also improves bothersome symptoms of obese men in mood, sense of well-being, vigor, vitality, and physical performance.^35^^,^^41^

Men with Type 2 DM (T2DM) frequently exhibit low TT levels, with up to 50% having symptoms of hypogonadism.^42^ In 355 men with T2DM (mean age 58.1 years), 20% and 31% had TT levels <8 nmol/L and 8–12 nmol/L, respectively, and 50% had free T < 0.255 nmol/L.^42^ In our study, 40.1% of ED men with T2DM had TT < 350 ng/dL (12.1 nmol/L). Lower T levels and greater severity of ED independently correlated with poorer physical function, social function, vitality, and decline in general health.^43^ TD and DM shared same mechanism and have similar clinical pictures in terms of prevalence and association with ageing and obesity.^44^ IR plays a central role in the development and progression of hyperglycemia, HT, dyslipidemia, and inflammation and provides the basis for a common understanding of these chronic diseases.^45^^,^^46^ Several mechanisms may account for the link between TD and DM and the potential role of TTh in DM, including the role of androgens in glucose transports, regulating glucagon-like peptide-1 receptor, maintaining pancreatic *β*-cell function, and suppressing inflammation.^47^ TTh increases skeletal muscle tissue and reduces abdominal obesity and nonesterified fatty acids, thereby improves IR.^20^ Many studies confer TTh improves IR and glycemic control in hypogonadal men with DM.^47–49^ Of hypogonadal obese men, subjects who received metformin or T injection for one year had HOMA-IR (Homeostasis Model Assessment for IR) decreased significantly by -2.4 (95% CI: -4.1–-0.8, *P* = .004) and -2.7 (95% CI: -4.3–-1.1, *P* = .001), respectively, compared with the placebo.^48^ In Testosterone for Diabetes Mellitus trial, TTh combined with lifestyle intervention for 2 years resulted in significant improvements in body composition, glucose regulation, and physical ability and decrease the risk of progression from pre-DM to overt T2DM by half vs. placebo (RR 0.51, 95% CI: 0.33–0.80).^49^ An 8-year study in hypogonadal men with pre-DM, the T-treated group had HbA1c decreased by 0.39 ± 0.03% (*P* < 0.001) and 90% had Hba1c < 5.7% whereas the T-untreated group had Hba1c increase by 0.63 ± 0.1% (*P* < 0.0001) and progressed to DM in 40.2%.^50^ Long-term TTh for 11-year in men with hypogonadism and T2DM, in T-treated group, 34.3% achieved sustained remission of their DM and 46.6% achieved normal glucose regulation and T- treated group had a lower mortality rate (7.3%) than the T-untreated group (29.2%).^47^ TTh is recommended potentially as an additional therapy for men with T2DM with hypogonadism.^47^ American Diabetes Association recommends screening for hypogonadism in men with T2DM or a BMI >30 or a waist circumference > 104 cm.^51^

Our findings also highlight the association between low TT levels and an increased risk of CAD in men over 50, as assessed by the Framinghm risk score. TD is associated with increased risk of all-cause mortality, mainly attributed to CV deaths.^52–54^ Longitudinal studies support an increased number of MACE in men with low T levels.^22^^,^^55^ ED significantly increased the risk of CVD, CAD, stroke, and all-cause mortality independent of conventional CV risk factors.^56^^,^^57^ In the Boston Area Community Health (BACH) survey with 965 men free of CVD, transient and persistent ED are associated with Framingham CVD risk and a greater increase risk in men younger under 50 years.^58^ Of 308 Korean men presented with sexual dysfunction, in the multiple linear regression analysis, a significant negative correlation was observed between the TT levels and the Framingham risk score.^59^ However, a longitudinal cohort study concluded that ED does not improve the prediction of who will and will not develop CVD beyond that offered by traditional risk factors.^60^ The identification of low T levels should alert the clinician thus identifying subjects with an increased CAD risk and MACE.^61^^,^^62^

Whether TTh has beneficial or detrimental effects on MACE remains controversial. The TRAVERSE trial, which assessed men with TD and high CV risk, found that TTh was not inferior to placebo regarding MACE incidence.^63^ The meta-analysis showed that TTh reduces overall mortality and CV morbidity in 15 pharmaco-epidemiological studies, whereas TTh had no clear effect on the incidence of CV events in 93 randomized-controlled trials (RCTs).^64^ A protective role of TTh on CV morbidity was observed when RCT trials enrolling obese patients.^64^ A 6-year follow-up study on 581 men with T2DM showed that low T levels predict an increase in all-cause mortality when controlled for covariates and TTh was associated with a reduced mortality of 8.4% compared with 19.2% (*P* = .002) in the untreated group.^65^ Testosterone exerts several beneficial effects to CV system including shortening QTc interval, improving glycemic control, anti-obesity, inducing vasodilatation and slowing atheroma progression.^66^ In spite of conflicting data of safety of TTh, review of the CV literature since 2015 showed that MACE were decreased with TTh in five studies, unchanged in 14, and increased in 0.^15^^,^^67^ In 77 hypogonadal men with a history of CVD, TTh for 8 years improves and preserves erectile function over prolonged periods with concurrent sustained improvements in cardiometabolic risk factors.^68^ Although no solid data support TTh reduces CV mortality, evidences suggest TTh improve a number of metabolic parameters associated with increased CV risk, such as fat mass, dyslipidemia, and IR.^15^

Regarding age-related T decline, our study found an average decrease of 17 ng/dL (0.6 nmol/L) per decade. This decline was independent of BMI. A study on non-obese men showed that all the TT, free T, and bioavailable T levels declined with age.^69^ The European Male Ageing Study (EMAS) study reported that TT levels did not decline significantly with age whereas free T did.^2^ Late-onset-hypogonadism is mixed with primary and secondary hypogonadism components attributed to varying pathophysiology and the comorbidities, suggesting T levels do not necessarily decline with age if men stay healthy.^70^

### Limitations

Several limitations existed in this study. The study participants all came from single institution and generalization of our study should be cautioned. Only TT levels were measured in this study whereas free T and bioavailable T were not measured. Besides, most of our participants had single sampling for TT levels whereas only 7.4% of them had repeat test of TT. The strengths of this study included a relatively big sample size and a comprehensive medical history and laboratory results collection.

## Conclusions

Our study confirms that TT levels are significantly associated with cardiometabolic risk factors, including obesity, DM, HT, and dyslipidemia in men. Measuring TT levels in men with ED presents an opportunity to improve overall health and reduce CV risk.
